# Carbon-Fixation Rates and Associated Microbial Communities Residing in Arid and Ephemerally Wet Antarctic Dry Valley Soils

**DOI:** 10.3389/fmicb.2015.01347

**Published:** 2015-12-09

**Authors:** Thomas D. Niederberger, Jill A. Sohm, Troy Gunderson, Joëlle Tirindelli, Douglas G. Capone, Edward J. Carpenter, S. Craig Cary

**Affiliations:** ^1^College of Marine and Earth Sciences, University of DelawareLewes, DE, USA; ^2^Wrigley Institute of Environmental Studies and Department of Biological Science, University of Southern CaliforniaLos Angeles, CA, USA; ^3^Romberg Tiburon Center, San Francisco State UniversityTiburon, CA, USA; ^4^International Centre for Terrestrial Antarctic Research, University of WaikatoHamilton, New Zealand

**Keywords:** CO_2_ fixation, Antarctic soils, primary production, Dry Valleys, microbial communities

## Abstract

Carbon-fixation is a critical process in severely oligotrophic Antarctic Dry Valley (DV) soils and may represent the major source of carbon in these arid environments. However, rates of C-fixation in DVs are currently unknown and the microorganisms responsible for these activities unidentified. In this study, C-fixation rates measured in the bulk arid soils (<5% moisture) ranged from below detection limits to ∼12 nmol C/cc/h. Rates in ephemerally wet soils ranged from ∼20 to 750 nmol C/cc/h, equating to turnover rates of ∼7–140 days, with lower rates in stream-associated soils as compared to lake-associated soils. Sequencing of the large subunit of RuBisCO (*cbbL)* in these soils identified green-type sequences dominated by the 1B cyanobacterial phylotype in both arid and wet soils including the RNA fraction of the wet soil. Red-type *cbbL* genes were dominated by 1C actinobacterial phylotypes in arid soils, with wetted soils containing nearly equal proportions of 1C (actinobacterial and proteobacterial signatures) and 1D (algal) phylotypes. Complementary 16S rRNA and 18S rRNA gene sequencing also revealed distinct differences in community structure between biotopes. This study is the first of its kind to examine C-fixation rates in DV soils and the microorganisms potentially responsible for these activities.

## Introduction

The McMurdo Dry Valleys (DV) of Antarctica represents one of the coldest, driest and most oligotrophic desert systems on Earth (Cary et al., 2010). Due to the lack of higher trophic levels, microorganisms dominate the arid DV soils (Cary et al., 2010), and as a result, community dynamics and ecological function are independent of other biological processes and are most likely directly coupled to the chemical and physical environment. This ecosystem therefore provides an extraordinary opportunity to examine metabolic adaptations that allow communities to function in extreme environments.

With the recent discovery of high microbial cell concentrations (5 × 10^5^ to 4 × 10^8^ g wet weight^-1^) and microbial diversity comparable to that of temperate soils(Cowan et al., 2002; Smith et al., 2006; Niederberger et al., 2008; Cary et al., 2010), there is great interest in resolving the sources, controls and turnover of carbon in these severely oligotrophic DV soils (Burkins et al., 2000, 2001; Barrett et al., 2005, 2006b, 2007; Elberling et al., 2006; Hopkins et al., 2006b, 2009; Cary et al., 2010; Feng et al., 2010). Soil organic carbon (SOC) concentrations in the bulk arid DV soils do not typically exceed 1.0 mg g^-1^, with concentrations being at least an order of magnitude higher in soils associated with ephemerally wetted lake and stream systems (Parsons et al., 2004; Barrett et al., 2006a; Elberling et al., 2006; Hopkins et al., 2006b, 2009; Ball et al., 2009; Cary et al., 2010; Feng et al., 2010). Wetted soils form in the DV during the summer months when temperatures become warm enough to melt lake ice edges and the surfaces of glaciers resulting in the formation of moats around the edges of lakes/ponds and short-lived (4–12 weeks) melt-water streams. Collectively, these form important hydrological links between glaciers and lakes (McKnight et al., 1999, 2007; Takacs-Vesbach et al., 2010). The wetted soils associated with these systems are well-documented hotspots of biogeochemical cycling and can contain dense microbial mat communities that bind the top 1–2 cm of soil together (Runkel et al., 1998; McKnight et al., 1999, 2004, 2007; Maurice et al., 2002; Gooseff et al., 2003). Mat communities in these ephemerally wet soils are typically cyanobacterial-, or moss-dominated and exhibit extremely patchy distribution (McKnight et al., 2004; Adams et al., 2006; Takacs-Vesbach et al., 2010). These communities survive the winter months in a desiccated state and, in some cases, are re-activated through hydration by summer melt-waters and over multiple wetting events can form large concentrations of responsive biomass (Vincent and Howard-Williams, 1986; McKnight et al., 1999).

In contrast to the high productivity wetted soils, SOC in bulk arid DV soils is hypothesized to originate from three major sources; (1) legacy deposits of ancient lake sediments, (2) allochthonous inputs from high productivity sites via wind transportation and (3) *in situ* CO_2_-fixation. As supported by laboratory-based studies, SOC turnover in DV soils has proven to be surprisingly rapid given the conditions, in the range of decades to ∼150 years (Burkins et al., 2001; Barrett et al., 2006b; Elberling et al., 2006; Hopkins et al., 2009; Tiao et al., 2012). It therefore seems unlikely that legacy deposits have survived to the present day, and if so, may only represent a minor or recalcitrant fraction of current SOC pools with more contemporary sources of C sustaining C-cycling in DV soils (Hopkins et al., 2006b; Feng et al., 2010).

Aeolian transport of mat detritus from high productivity sites has also been hypothesized to be an important source of organic C to bulk arid DV soils (Elberling et al., 2006) and a proven facilitator of soil respiration (Hopkins et al., 2006a). However, aeolian transport has been estimated to be an insignificant method of carbon delivery (0.01–7 g C m^-2^ year^-1^) and may only be relevant to regions in close proximity to lake systems (Lancaster, 2002; Barrett et al., 2006b). Independent studies have also shown that the natural stable isotopic (^13^C and ^15^N) abundances of soil organic matter (SOM) differ between high and low productivity sites, with SOC isotopic signatures from remote locations (i.e., at large distance from wet sources and at higher elevation) resembling endolithic sources, and sites closer to high productivity sites and at lower elevation resembling lacustrine signatures (Burkins et al., 2000, 2001; Hopkins et al., 2009). Most recently, through the use of both gas chromatography/mass spectrometry and nuclear magnetic resonance spectroscopy, Feng et al. (2010) have shown that certain SOM compounds differ between bulk arid soils and microbial mats associated with a nearby lake, suggesting either a fast turnover of lake derived material in nearby arid soils or insignificant aeolian distribution (Feng et al., 2010). *In situ* CO_2_-fixation (primary productivity) has therefore been hypothesized to be the most consistent source of C, appearing to make the largest contribution to SOC and replenishing C stocks in DV soils (Burkins et al., 2000, 2001; Hopkins et al., 2009). However, C-fixation rates and the microorganisms responsible for these activities in DV soils remains largely unknown with a recent DNA-based study (Chan et al., 2013) of DV biotopes indicating that autotrophic functionalities are present in the endemic biota encompassing members of the Cyanobacteria, Archaea, Actinobacteria, and Proteobacteria.

The most common CO_2_-fixation pathway for chemo- and phototrophs is the reductive pentose phosphate/Calvin–Benson–Bassham (CBB) cycle. Ribulose-bisphosphate carboxylase (RuBisCO; EC 4.1.1.39) is a key enzyme responsible for the fixation of CO_2_ in this pathway (Tourova and Spiridonova, 2009) and exists as two major forms, I and II, that share ∼25–30% amino acid similarity. Form I RuBisCO has eight large subunits (encoded by the *cbbL* gene) and eight small subunits (encoded by the *cbbS* gene). Based on phylogenetic analyses the large subunit can be further divided into two independent ‘green’ and ‘red’ types as defined by amino acid sequence identities. The green-type has two variants: IA occurring in several proteobacteria and IB occurring in plants, green algae, and cyanobacteria. The red-type also has two variants: IC, specific to α- and β-proteobacteria and ID to non-green algae (Alfreider et al., 2009; Tourova and Spiridonova, 2009). Form II RuBisCO consists of two large subunits encoded by the *cbbM* gene (Shively et al., 1998). The *cbbL* and *cbbM* genes are routinely used as molecular markers for the identification of autotrophs in natural microbial communities (Watson and Tabita, 1997; Giri et al., 2004; van der Wielen, 2006; Alfreider et al., 2009; Videmšek et al., 2009), due to sequence conservation, its essential function in C-fixation and the large number of RuBisCO gene sequences in public databases. However, whilst RuBisCO sequences do provide valuable phylogenetic insight into the identity of C-fixers, RuBisCO taxonomic groupings do not always concur with 16S rRNA gene phylogeny, most likely the result of horizontal gene transfer and gene loss or duplication events (Spiridonova et al., 2004; Tourova and Spiridonova, 2009).

C-fixation is hypothesized to be a highly important process in DV soils, perhaps representing the major source of C in the bulk arid soils. However, rates of C-fixation in both arid and high productivity soils of the DV are unknown, and the microorganisms responsible for these activities remain unidentified. The objective of this study was therefore to: (1) estimate rates of C-fixation, as measured by ^13^CO_2_ uptake in contrasting wet and dry DV soil habitats and (2) identify microbial community structure and identify C-fixers via complementation of 16S rRNA and 18S rRNA gene sequencing with RuBisCO gene sequencing. Results from this study revealed distinct differences in community structure between both arid and wetted DV soils biotopes. Moreover, rates of C-fixation detected in both biotopes with low levels of SOC were higher than expected. Therefore, these collective results lend further credence to the hypothesized turnover rates of C in DV soils.

## Materials and Methods

### Site Description and Sample Collection

Sites and sampling methods are as described previously (Niederberger et al., 2012). In short, a transect-based sampling approach was utilized consisting of three or four sampling points originating from site 1 defined as a “wet” zone (soils with overlying stream, lake or pond water) extending through a hyporheic zone containing obvious microbial mats to the final site (i.e., site 3 or 4) situated in a typical arid DV mineral soil. Gravimetric water content of soil samples were measured as described previously (Niederberger et al., 2012).

### Measurement of CO_2_-fixation Rates

CO_2_ fixation was measured using a stable isotope enrichment method modeled after Finzi-Hart et al. (2009). Mats were sampled with a cut-off 5 ml plastic syringe and four 1 cm deep cores placed in a 27 ml glass serum bottle. Serum bottles were completely filled with water from the sampling site and gas bubbles removed before sealing with a rubber septa and crimp seal. A total of 10 μl of 0.233 M NaH^13^CO_3_ was then added and samples incubated for 24 h at *in situ* conditions. After incubation, bottles were opened and the contents poured into 50 ml centrifuge tubes. Samples were rinsed three times by washing with stream water, centrifuging at low speed and pouring off the supernatant, and then dried at 60°C. Samples were then homogenized in a mortar and pestle, weighed into aluminum foil cups and folded into small pellets using forceps. N and C content and stable isotope mass ratio (d^15^N, and d^13^C) were determined at the UC Davis Stable Isotope Facility Davis (Davis, CA 95616, USA). Values were corrected using internal standards and CO_2_ fixation rates calculated as detailed previously (Montoya et al., 1996).

### Nucleic Acid Isolation, cDNA Synthesis from mRNA, and Polymerase Chain Reaction (PCR)

Nucleic acid (DNA and RNA) isolation, cDNA synthesis and confirmation of DNA removal in RNA extracts and DNase treated extracts undertaken as described previously (Niederberger et al., 2012). RuBisCO form I *cbbL* green- and red-type genes were PCR amplified utilizing respective primer pairs RubIgF:RubIgR and RubIrF:RubIrR corresponding to positions 571–1382 and 196–1016 of the *cbbL* gene as outlined by Spiridonova et al. (2004). Commonly utilized primer pair, cbbL595F:cbbL1387R (Elsaied and Naganuma, 2001; Giri et al., 2004), was also tested to amplify the form I *cbbL* green-type gene; however, non-specific banding was observed within electrophoretic profiles (results not shown). The RuBisCO from II *cbbM* gene was PCR amplified utilizing a nested approach with primer pairs RuIIF1:RuIIR3 and RuIIF2:RuIIR2 as outlined by Spiridonova et al. (2004).

### Gene Cloning and Restriction Fragment Length Polymorphism (RFLP) Analyses

RuBisCO PCR amplicons were excised from ethidium stained 2% agarose TAE gels and purified using the GenElute^TM^ gel extraction kit (SIGMA) and ligated (pCR4-TOPO vector; Invitrogen), transformed (One Shot TOP10 chemically competent *Escherichia coli*; Invitrogen) and clones selected as described previously (Niederberger et al., 2012). Inserts from ∼30 clones of each 96 clone library were sequenced and the remainder of the clones screened by RFLP using restriction enzymes (*Hae*III and *Rsa*I; New England BioLabs) as commonly used for partial length *cbbL* amplicons (Alfreider et al., 2009). Due to a high frequency of *Hae*III cut sites and the subsequent small DNA fragments (∼ <50 bp), visualization and comparison between samples within agarose gels was difficult (also confirmed through simulated *in silico* restriction endonucluease digestion of pre-sequenced clones). Therefore, RFLP was performed with *Rsa*I alone and representative clones of each RFLP type sequenced. *cbbL* amino-acid sequences are deposited as accession numbers KP836071 to KP836108 in the NCBI GenBank database *cbbL* gene sequences and 16S rRNA and 18S rRNA gene sequences are deposited in the Knowledge Network for Biocomplexity^1^ under identifier ID: knb.756.1.

### cbbL Gene Analyses

Sequences were aligned using ClustalW (Thompson et al., 1994) and a PHYLIP output file used to construct a Jukes-Cantor corrected distance matrix by the DNADIST program of PHYLIP^2^ and operational taxonomic units (OTUs) defined and rarefaction analyses undertaken using the DOTUR program (Schloss and Handelsman, 2005). Representative *cbbL* gene sequences (90% sequence similarity) were aligned and translated within the Geneious software environment^3^ and aligned to translated *cbbL* genes obtained from the NCBI GenBank database. The alignment was manually checked and a phylogenetic tree constructed using the Jukes-Cantor genetic distance model and the Neighbor-joining method with 1000 bootstrap re-samplings.

### Amplicon Pyrosequencing, Processing, and Analyses

Tag-encoded FLX (Roche) amplicon pyrosequencing of the V1–V3 regions of the 16S and 18S rRNA gene was performed on DNA extracts by Research and Testing Laboratories (Lubbock, TX, USA^4^). Resulting data were then processed using the Quantitative Insights Into Microbial Ecology (QIIME) toolkit (Caporaso et al., 2010). In brief, rRNA gene sequences were quality trimmed (QIIME defaults; >200 bp), split according to barcoded tags and sequences binned into operational taxonomic units (OTU) at 97 and 95% for bacteria and eukaryotes, respectively. Following quality trimming, a total of 7640 and 7163 partial length (>200 bp) 16S rRNA gene sequences were obtained for ML1–2 and ML1–4 respectively. Bacterial taxonomic assignment was undertaken on all quality trimmed 16S rRNA gene sequences using the online RDP classifier tool (at 80 confidence level) and associated RDP database (Cole et al., 2009). Eukaryotic taxonomic assignment was undertaken on a representative sequence from each OTU using the Basic Local Alignment Search Tool (BLAST) within the QIIME toolkit against the SILVA 18S rRNA gene database (Pruesse et al., 2007) as obtained from mothur (Schloss et al., 2009). Bacterial 16S rRNA gene rarefaction analyses and library comparisons (LIBCOMPARE) were performed using the tools within the online RDP pyrosequencing pipeline (Cole et al., 2009) and 18S rRNA gene rarefaction within mothur (Schloss et al., 2009).16S rRNA gene and 18S rRNA gene sequences are deposited within the Knowledge Network for Biocomplexity as stated above.

## Results

A total of four transects in the vicinity of Miers Valley were utilized as part of the study, including both lake- and stream-associated sites (Supplementary Table S1). Carbon-fixation rates were higher at lake-associated soils (2.15–751.29 nmol C/cc/h) as compared to stream systems (below detection limits to 15.83 nmol C/cc/h). Differences in C-fixation rates between dry and wetted soils was only apparent for lacustrine soils with rates for arid soils (<5% water content) ranging from undetected to 11.83 nmol C/cc/h as compared to wetted soils (12.52–751.29 nmol C/cc/h).

The ML1 transect on the northern shore of Miers Lake was chosen as a representative transect for the in-depth identification of C-fixers and microbial communities between contrasting wet (ML1–2, ∼23% moisture content) and dry soils (ML1–4, ∼2% moisture content). This transect was chosen due to the noticeable differences in C-fixation rates between wetted and arid soils, and because the N_2_-fixing microorganisms and nitrogenase activities of the soils have previously been described (Niederberger et al., 2012).

Both form I green- and red-type *cbbL* genes were detected in total DNA extracts from wet (ML1–2) and dry (ML1–4) sites (detection of *cbbL* genes by PCR is summarized in Supplementary Table S1). Amplicons of the green-type *cbbL* gene were faintly detectable by electrophoresis from cDNA in ML1–2, with an additional 10 thermocycles providing sufficient amplicon concentrations for cloning purposes; however, the green-type *cbbL* gene was not detected in cDNA from the arid (ML1–4) site. The red-type *cbbL* gene was not detected in cDNA from either ML1–2 or ML1–4. The form II *cbbM* gene was also detectable in DNA extracts; but not in the corresponding cDNA preparations. Because the expressed *cbbM* gene was not detected in these samples, *cbbL* was utilized to identify the autotrophic microorganisms inhabiting these sites.

The total number of *cbbL* gene clones from each library ranged between 61 and 80 (Supplementary Table S1) with the exception of ML1–2 cDNA. This library contained only 44 sequences due to the presence of large number (49) of sequences closely related to 23S rRNA genes (results not shown), attributed to non-specific PCR amplification. Although low numbers of *cbbL* clones were sequenced, rarefaction (90%) analyses indicate that, for all samples, the green-type sequences were well-represented whilst red-type sequences were under-represented (Supplementary Information: Supplementary Figure S1).

**FIGURE 1:**
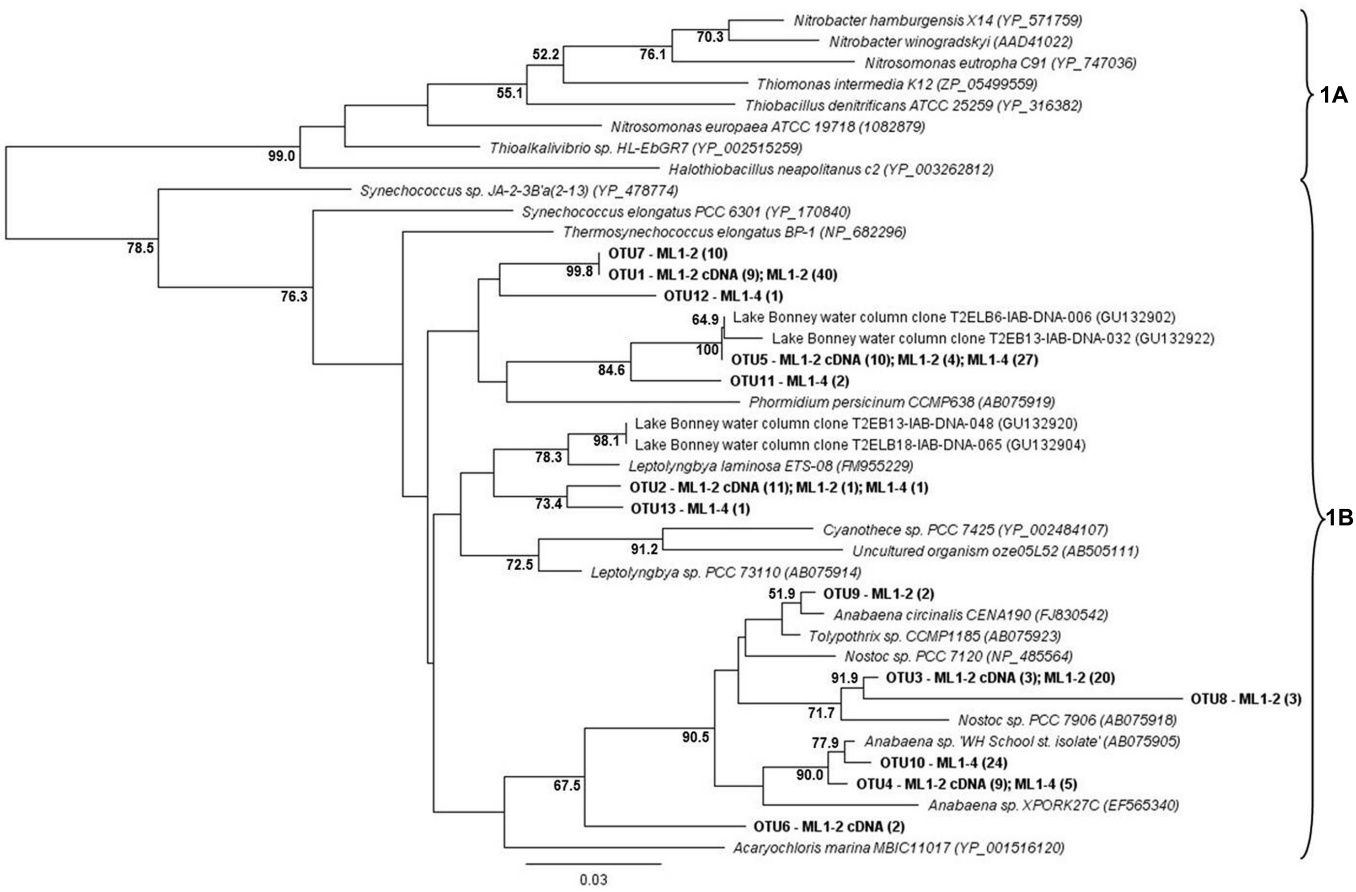
**Neighbor joining tree showing the phylogenetic relationships of translated green-type *cbbL* sequences as based on 127 amino-acid residues**. The tree was constructed using the Jukes-Cantor distance model. *Sinorhizobium meliloti* 1021 (NP_436731) was used as an out-group and has been removed from the tree. Bootstrap supports are indicated as percentages (>50%) of 1000 replicates.

**FIGURE 2:**
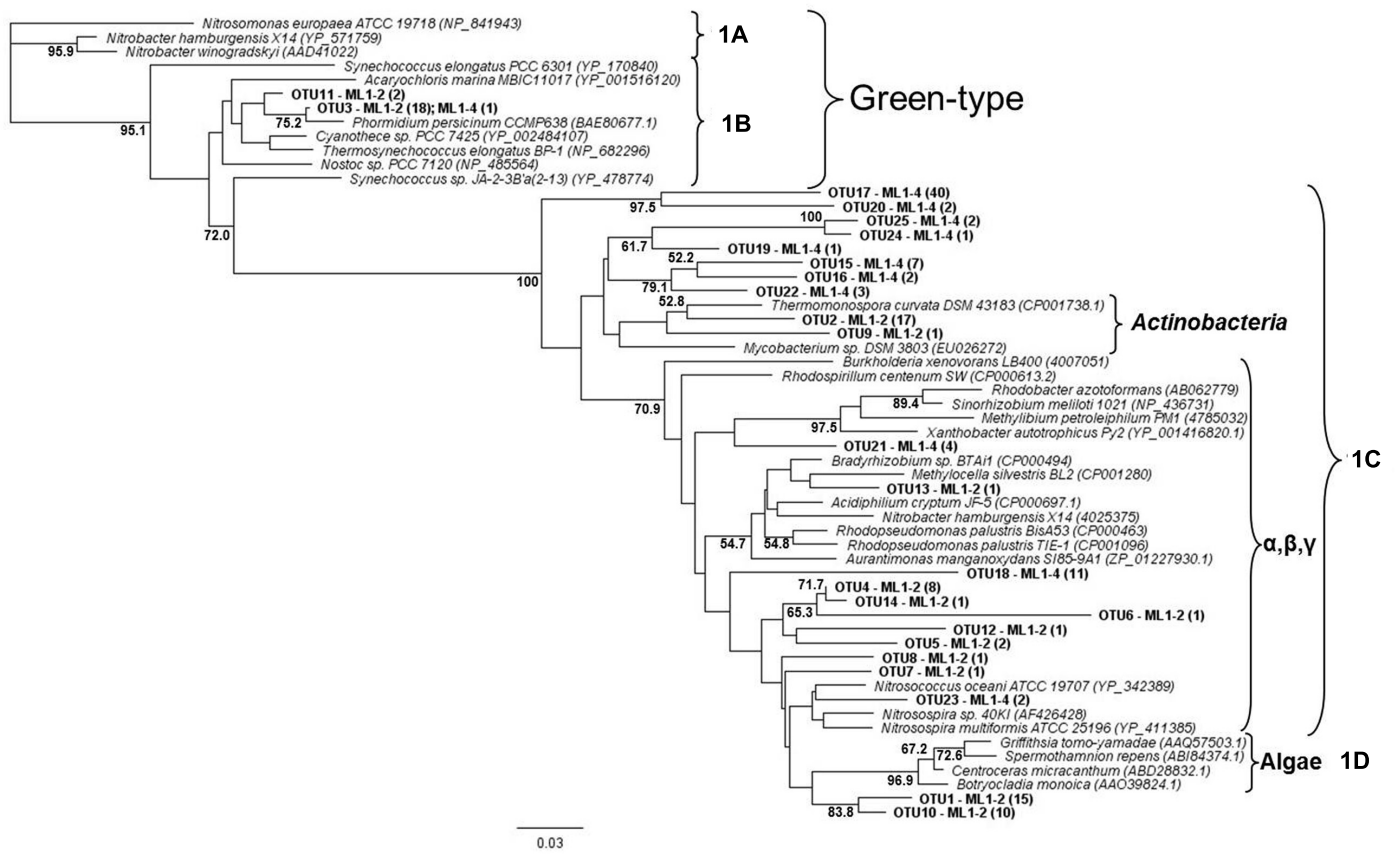
**Neighbor joining tree showing the phylogenetic relationships of translated red-type *cbbL* sequences as based on 120 amino-acid residues**. The tree was constructed using the Jukes-Cantor distance model and green-type sequences was used as an out-group. Bootstrap supports are indicated as percentages (>50%) of 1000 replicates.

For both the wet and dry samples, 100% of the green type *cbbL* phylotypes grouped within the 1B variant of cyanobacterial-related phylotypes (**Figure 1**), i.e., the green-type 1A variant was not detected. Diversity levels were similar for all sample types, 7, 6, and 7 OTUs for ML1–2, ML1–2 cDNA, and ML1–4, respectively and the majority of the sequences were most closely related to clones from the water column of Lake Bonney in the DV (**Figure 1**). Unexpectedly, the red-type clone libraries also contained 1B variant green-types (**Figure 2**). However, these 1B variant sequences were not added to the green-type phylogenetic tree as the red-type primers amplify a different region of the green-type *cbbL* gene than targeted by the green-type primers. In contrast to the green-type library, the wet and the dry samples differed considerably in the red-type library. For the dry ML1–4 soil, the red-type library was dominated by 1C variant phylotypes related to the actinobacteria (76%, **Figure 2**), with the remainder related to the proteobacterial 1C variant clade while the actinobacterial and proteobacterial 1C variants made up only 23 and 20% of the total phylotypes detected in the wet ML1–2 soil and 1D algal signatures were numerically dominant in this sample (32%, **Figure 2**).

**FIGURE 3:**
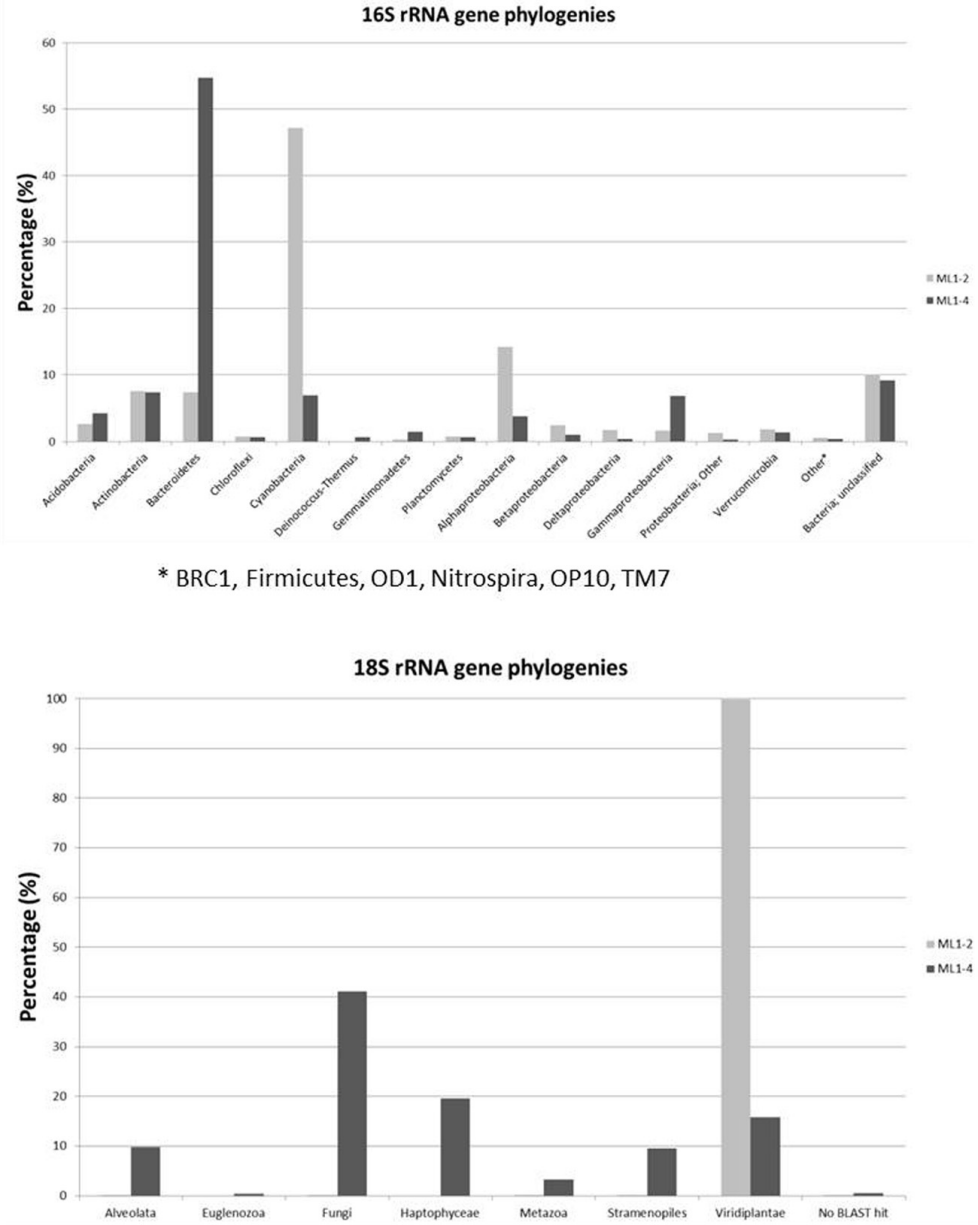
**Bacterial and Eukaryotic phylogeny of 16S rRNA and 18S rRNA genes from wet (ML1–2) and dry (ML1–4) Miers Lake soil sites**.

Diversity plateaus within 16S rRNA gene based rarefaction plots indicate sufficient representation (Supplementary Information: Supplementary Figure S2), with a lower total diversity level for the arid ML1–4 site. Taxonomic affiliations of the 16S rRNA sequences are presented in **Figure 3**. Noticeable differences between the wet (ML1–2) and arid (ML1–4) sites include higher concentrations of cyanobacteria and alphaproteobacteria for ML1–2 (i.e., 47 and 14%, respectively and 7 and 4% for ML14), and higher concentrations of *Bacteroidetes* and gammaproteobacteria for ML1–4 (55 and 7%, respectively vs. 7 and 2% for ML1–2). Various significant (as supported by e-scores) differences in taxonomic representation were observed between the wet and dry sites using the online RDP LIBCOMPARE tool at an 80% confidence level. The most significant (<1E^-12^) are listed in Supplementary Table S2 and include the presence of *Gillisia* in dry ML1–4 soil (∼27% of total *Bacteroidetes*) and the absence of this genus in wet ML1–2 soil, a high contribution (∼59%) of *Streptophyta* in the total cyanobacteria detected in the wet ML1–2 soil with this genus being unobserved in the arid ML1–4 site. The GpI group of cyanobacteria were also well-represented in the wet ML1–2 soil (∼26% of total detected cyanobacteria) with less than 1% representation in dry ML1–4 soil, and interestingly, the majority (∼91%) of the cyanobacteria detected in the dry ML1–4 soil were related to the GpIV group, with this group only making up 6% of wet-associated cyanobacterial signatures. Although only minor components of the dry ML1–4 soil (<1%), members of the Deinococcus-Thermus group were not detected in the wet ML1–2 soil.

A total of 14,642 and 2,958 quality trimmed 18S rRNA gene sequences were obtained for ML1–2 and ML1–4 respectively. Rarefaction analyses at 90% sequence cut-off, indicate sufficient sampling for both ML1–2 and ML1–4 with higher diversity levels observed at the dry ML1–4 site (Supplementary Figure S3). Phylogenetic analyses indicate that wetted ML1–2 soil was almost completely dominated (>99%) by Virdiplantae, with 99.8% of these sequences most closely related to the moss Ephemerum (**Figure 3**). For the dry soil, a diverse assemblage of eukaryotes was observed including members of various phyla (**Figure 3**).

## Discussion and Conclusion

Dry Valley soils are severely carbon-limited and can be considered some of the most oligotrophic on the planet (Cary et al., 2010). Current literature has highlighted the importance of C-fixation as a means of consistent replenishment of organic carbon in DV soils (Parsons et al., 2004; Hopkins et al., 2009; Feng et al., 2010); however, the extent that DV soil microbial communities undertake C-fixation or their reliance on external sources of C remains unknown. This study therefore provides important insights into this fundamental biogeochemical cycle and the understanding of carbon transformations in DV soils by focusing on communities found in the Miers Valley. Autotrophy is an energetically expensive process, which is usually slow and under strict control (Alfreider et al., 2009), especially in limited conditions such as soils of the DV. Therefore, complementary stable isotope- and mRNA-based methods were applied to resolve activities and to identify the associated active microbial component.

As expected, carbon fixation rates were typically higher wetter soils as compared to arid soils (>5% moisture) with rates being considerably lower in stream systems as compared to the lake-associated soils. This is most likely caused by the transient nature of the streams making the establishment of permanent microbial communities difficult as evidenced by the formation of thick mats on lake edges but not in the Miers Valley streams. The transects involved in this study have also been described in a previous study investigating nitrogen-fixation activities in these soils (Niederberger et al., 2012).

Few studies have reported carbon-fixation rates in the DV, with rates in the bulk arid soils being considered extremely low, ranging from 1 to 20 g C m^-2^ year^-1^ (Friedmann, 1993; Novis et al., 2007; Cary et al., 2010). Rates of carbon addition to DV soils are also hypothesized to be less than or equal to respiration rates (∼ <6.5 g C m^-2^ year^-1^) otherwise soil carbon reservoirs would already be depleted (Burkins et al., 2001). In this study, C turnover rates in the ephemerally wet soils ranged between ∼7 and 140 days and equated to micrograms of C-fixed per cubic centimeter on a daily basis in both wetted and arid biotopes. These rates fall within the same range (3.22 μg C L^-1^ day^-1^) as recently measured in the water column of Lake Bonney situated in the Antarctic DV (Kong et al., 2012a). C-fixation rates reported in this study are well-above previous estimates; however, it is important to note that rates were measured during the high productivity summer months associated with warmer temperatures, wetter soils and a longer photo-period, and focused specifically on microbial mats. Therefore, at least during the Polar summer period, autotrophs play a major role in carbon replenishment of DV soils and further corroborate suggestions that as opposed to the reliance of carbon from high productivity soils from aeolian redistribution (Burkins et al., 2000, 2001; Barrett et al., 2006c; Hopkins et al., 2009; Feng et al., 2010), *in situ* CO_2_-fixation may be the largest contributor to SOC in DV soils (Burkins et al., 2000, 2001; Hopkins et al., 2009).

The cyanobacterial-related form 1B *cbbL* gene was the only phylotype detected in the green-type sequences for both the wet and dry soils, with the 1A proteobacterial-related phylotype remaining undetected. These cyanobacterial sequences from both the wet and dry sites were related to sequences from Lake Bonney in Taylor Valley, a larger and more northern Valley than the Miers (Kong et al., 2012b). The 1B green-type phylotype was the only *cbbL* gene expressed in the wet sample, indicating that at the time of sampling, cyanobacteria were the major contributors to the observed C-fixation there. Although C-fixation rates were measureable in some arid soil samples, expression of RuBisCO genes was not seen, and therefore, the members directly responsible for these activities remain unidentified. Expression of red-type sequences was also not seen in the wet or dry soils, but the presence of the genes in DNA isolated from these sites shows that there were other organism present besides caynobacteria that have the potential to fix carbon. The wet soil contained both 1C- (both actinobacterial and proteobacterial) and 1D-related sequences, with algal (1D) dominance (32%). Similarly 1D-related sequences have been proven to dominate the water column of Lake Bonney in the DV (Kong et al., 2012b). Proteobacterial red-type signatures in the wetted soil were most closely related to nitrifying bacteria, a group of chemolithoautotrophs that oxidize either ammonia or nitrite and are typically present in areas of high ammonia concentrations. While we did not attempt to measure nitrification in this study, it is known to occur in DV soils, with highest rates at wetted lake margins (Hopkins et al., 2006a), suggesting that some of the measured C-fixation could be based on chemical- rather light-based energy. Red-type gene sequences were not shared between wet and dry soils; the bulk arid soil was dominated by actinobacterial-related 1C signatures (76%) with algal (1D) phylotypes absent. 16S rRNA gene results did not reflect the difference in actinobacterial *cbbL* gene presence in dry vs. wet soils, with a similar percentage of actinobacterial signatures in both biotopes as was also noted in a previous study comparing high and low DV productivity soils (Niederberger et al., 2008) with 18S rRNA gene sequencing indicating an almost complete dominance of wetted soils by Virdiplantae. Babalola et al. (2009) investigate and describe in depth, Actinobacteria in DV soils with various molecular-based studies proving that both *Actinobacteria* and *Bacteroidetes* are commonly dominant members of arid soils including the DV (Fierer et al., 2007; Niederberger et al., 2008; Pointing et al., 2009; Lee et al., 2012; Tiao et al., 2012; Bottos et al., 2014); therefore, it is not surprising that this group of organisms have C-fixation capabilities thereby permitting their subsistence in these severely oligotrophic soils. In fact, an important study by Chan et al. (2013) has reported the presence of RuBisCo signatures in Antarctic DV habitats specifically form I from cyanobacteria and forms II and III indicated as being from Archaea, *Actinobacteria*, and Proteobacteria. These results also suggest a significant capability in chemoautotrophy in these habitats.

The rates of C-fixation in both arid and wetted DV soils reported in this study coupled with documented low levels of SOC (Parsons et al., 2004; Barrett et al., 2006a; Elberling et al., 2006; Hopkins et al., 2006b, 2009; Ball et al., 2009; Cary et al., 2010; Feng et al., 2010) lends further credence to the hypothesized high turnover rates of C in DV soils. Therefore, at least in the summer months, *in situ* autotrophic C-fixation can replenish soil SOC levels with arid soils most likely dominated by actinobacterial C-fixers with a more diverse microbial community in wetted soils dominated by cyanobacterial-related activity. The recent discovery of genetically localized communities between valleys also indicates that these communities maybe endemic and that inter-valley aeolian-based redistribution maybe negligible (Lee et al., 2012). If this holds true, communities in these distinct biotopes cannot rely on consistent external sources of carbon and must be adapted to exist under these extreme dry and nutrient-limited conditions.

## Conflict of Interest Statement

The authors declare that the research was conducted in the absence of any commercial or financial relationships that could be construed as a potential conflict of interest.

## Footnotes

1 https://knb.ecoinformatics.org

2 www.phylip.com

3 www.geneious.com

4 www.researchandtesting.com
