# Supplementary material for: Carbon-Fixation Rates and Associated Microbial Communities Residing in Arid and Ephemerally Wet Antarctic Dry Valley Soils
Source: Front Microbiol. 2015 Dec 9;6:1347. doi: 10.3389/fmicb.2015.01347 (PMC4673872; doi:10.3389/fmicb.2015.01347)
Supplement: Supplementary file 1 [file Table_1.DOCX]

TABLE S1 | Description of samples collected along various transects from a wet origin to arid soils and associated moisture, distance, and C-fixation data.

| **Site** | **Location** | **Location** | **Distance from wet source (m)** | **Water content (%)** | **C-fixation rates (s.d.*)**  **(nmol C/cc**/h)** |
| --- | --- | --- | --- | --- | --- |
| MS1-1 | Miers Stream | S78°05.615ˈ  E163°49.912ˈ | origin | submerged | 1.02 (0.31) |
| MS1-2 |  |  | 9.6 | 4.5 | n.d. |
| MS1-3 |  |  | 13.5 | 19.2 | n.d. |
| MS1-4 |  |  | 20.0 | 3.2 | n.d. |
| MS2-1 | Miers Stream | S78°05.745ˈ  E163°45.885ˈ | origin | 17.78 | n.d. |
| MS2-2 |  |  | 3.0 | 5.84 | 12.52 (2.07) |
| MS2-3 |  |  | 4.5 | 2.29 | 11.83 (5.54) |
| MS2-4 |  |  | 6.5 | 0.27 | n.d. |
| ML1-1 | Miers Lake (Northern shore) | S78°05.615ˈ  E163°49.912ˈ | origin | submerged | 87.81 (47.87) |
| ML1-2 |  |  | 3.0 | 22.73 | 751.29 |
| ML1-3 |  |  | 3.7 | 14.54 | 87.35 (67.53) |
| ML1-4 |  |  | 8.3 | 2.1 | 2.15 |
| ML2-1 | Miers Lake (Northern shore) | S78°05.643ˈ  E163°52.026ˈ | origin | 21.48 | 575.63 (340.92) |
| ML2-2 |  |  | 2.5 | 31.44 | 166.94 (89.11) |
| ML2-3 |  |  | 5.0 | 12.09 | 222.61 |

s.d. = standard deviation; n.d. = not detected

*standard deviation not calculated for results based on a single measurement.

**cubic centimeters
