# Supplementary material for: Carbon-Fixation Rates and Associated Microbial Communities Residing in Arid and Ephemerally Wet Antarctic Dry Valley Soils
Source: Front Microbiol. 2015 Dec 9;6:1347. doi: 10.3389/fmicb.2015.01347 (PMC4673872; doi:10.3389/fmicb.2015.01347)
Supplement: Supplementary file 2 [file Table_2.DOCX]

**Table S2.** Significant differences (results <1E^-12^ shown) in taxonomic representation between wet (ML1-2) and dry (ML1-4) soils as classified and compared at 80% confidence using the online RDP LIBCOMPARE tool. The number of 16S rRNA gene sequences for each taxonomic group at each soil site are presented and the associated significance (E-score).

| **Rank** | **Name** | **ML1-2** | **ML1-4** | **Significance** |
| --- | --- | --- | --- | --- |
| genus | *Gillisia* | 0 | 1040 | 0.00E+00 |
| genus | *Streptophyta* | 2145 | 0 | 0.00E+00 |
| family | *Family I* | 929 | 1 | 6.41E-265 |
| genus | *GpI* | 929 | 1 | 6.41E-265 |
| family | *"Cyclobacteriaceae"* | 1 | 526 | 3.96E-164 |
|  | *unclassified "Cyclobacteriaceae"* | 0 | 454 | NA |
| family | *Caulobacteraceae* | 191 | 5 | 9.01E-48 |
| order | *Caulobacterales* | 191 | 5 | 9.01E-48 |
| genus | *Amaricoccus* | 138 | 1 | 1.61E-38 |
| genus | *Brevundimonas* | 154 | 5 | 1.35E-37 |
| genus | *Patulibacter* | 1 | 116 | 1.60E-35 |
| family | *Patulibacteraceae* | 1 | 116 | 1.60E-35 |
| family | *Chromatiaceae* | 0 | 105 | 7.66E-34 |
| order | *Chromatiales* | 0 | 105 | 7.66E-34 |
| genus | *Rheinheimera* | 0 | 105 | 7.66E-34 |
| family | *Pseudomonadaceae* | 0 | 85 | 1.55E-27 |
| order | *Pseudomonadales* | 0 | 85 | 1.55E-27 |
| genus | *Pseudomonas* | 0 | 83 | 6.60E-27 |
| family | *Microbacteriaceae* | 102 | 3 | 1.17E-25 |
| genus | *Lysobacter* | 5 | 96 | 1.69E-24 |
| order | *Rhodospirillales* | 105 | 5 | 2.54E-24 |
| genus | *Nitriliruptor* | 0 | 74 | 4.54E-24 |
| family | *Nitriliruptoraceae* | 0 | 74 | 4.54E-24 |
| order | *Nitriliruptorales* | 0 | 74 | 4.54E-24 |
| family | *Acetobacteraceae* | 104 | 5 | 4.71E-24 |
| genus | *Aquiflexum* | 0 | 71 | 4.01E-23 |
| genus | *Spirosoma* | 60 | 0 | 6.00E-18 |
| genus | *Roseomonas* | 78 | 5 | 3.55E-17 |
| suborder | *Propionibacterineae* | 64 | 2 | 2.28E-16 |
| family | *Hyphomicrobiaceae* | 49 | 0 | 8.67E-15 |
| family | *Cryomorphaceae* | 1 | 48 | 1.88E-14 |
| phylum | *"Deinococcus-Thermus"* | 0 | 43 | 2.69E-14 |
| order | *Deinococcales* | 0 | 43 | 2.69E-14 |
| class | *Deinococci* | 0 | 43 | 2.69E-14 |
| genus | *Flavisolibacter* | 0 | 43 | 2.69E-14 |
| genus | *Truepera* | 0 | 43 | 2.69E-14 |
| family | *Trueperaceae* | 0 | 43 | 2.69E-14 |
|  | *unclassified Microbacteriaceae* | 56 | 2 | NA |
| phylum | *"Bacteroidetes"* | 560 | 3917 | 6.00E-14 |
| order | *"Flavobacteriales"* | 64 | 2320 | 6.00E-14 |
| phylum | *"Gemmatimonadetes"* | 23 | 107 | 6.00E-14 |
| phylum | *"Proteobacteria"* | 1613 | 892 | 6.00E-14 |
| class | *"Sphingobacteria"* | 431 | 887 | 6.00E-14 |
| order | *"Sphingobacteriales"* | 431 | 887 | 6.00E-14 |
| subclass | *Actinobacteridae* | 479 | 243 | 6.00E-14 |
| order | *Actinomycetales* | 479 | 165 | 6.00E-14 |
| class | *Alphaproteobacteria* | 1088 | 274 | 6.00E-14 |
| family | *Chloroplast* | 2219 | 13 | 6.00E-14 |
| phylum | *Cyanobacteria* | 3606 | 498 | 6.00E-14 |
| family | *Cytophagaceae* | 115 | 15 | 6.00E-14 |
| class | *Deltaproteobacteria* | 132 | 28 | 6.00E-14 |
| family | *Family IV* | 226 | 452 | 6.00E-14 |
| class | *Flavobacteria* | 64 | 2320 | 6.00E-14 |
| family | *Flavobacteriaceae* | 60 | 2228 | 6.00E-14 |
| class | *Gammaproteobacteria* | 122 | 490 | 6.00E-14 |
| family | *Gemmatimonadaceae* | 23 | 107 | 6.00E-14 |
| order | *Gemmatimonadales* | 23 | 107 | 6.00E-14 |
| class | *Gemmatimonadetes* | 23 | 107 | 6.00E-14 |
| genus | *Gemmatimonas* | 23 | 107 | 6.00E-14 |
| genus | *GpIV* | 226 | 452 | 6.00E-14 |
| order | *Rhizobiales* | 183 | 48 | 6.00E-14 |
| subclass | *Rubrobacteridae* | 50 | 221 | 6.00E-14 |
| order | *Solirubrobacterales* | 45 | 199 | 6.00E-14 |
| family | *Sphingomonadaceae* | 249 | 22 | 6.00E-14 |
| order | *Sphingomonadales* | 284 | 81 | 6.00E-14 |
| family | *Xanthomonadaceae* | 96 | 222 | 6.00E-14 |
| order | *Xanthomonadales* | 96 | 222 | 6.00E-14 |
|  | *unclassified "Bacteroidetes"* | 65 | 710 | NA |
|  | *unclassified Actinomycetales* | 145 | 30 | NA |
|  | *unclassified Cyanobacteria* | 230 | 27 | NA |
|  | *unclassified Flavobacteriaceae* | 27 | 1092 | NA |
|  | *unclassified Sphingomonadaceae* | 164 | 9 | NA |
| genus | *Pseudoxanthomonas* | 2 | 49 | 1.27E-13 |
| genus | *Hymenobacter* | 44 | 0 | 2.37E-13 |
| genus | *Sphingomonas* | 72 | 7 | 2.58E-12 |
| order | *Myxococcales* | 98 | 19 | 5.24E-12 |
| family | *Nocardioidaceae* | 44 | 1 | 5.39E-12 |
