# Supplementary material for: Carbon-Fixation Rates and Associated Microbial Communities Residing in Arid and Ephemerally Wet Antarctic Dry Valley Soils
Source: Front Microbiol. 2015 Dec 9;6:1347. doi: 10.3389/fmicb.2015.01347 (PMC4673872; doi:10.3389/fmicb.2015.01347)
Supplement: Supplementary file 3 [file Data_Sheet_1.DOCX]

**Figure 1.** Rarefaction at 90% identity of *cbbL* genes detected in wet (ML1-2) and arid (ML1-4) soil sites.
