# Supplementary material for: Carbon-Fixation Rates and Associated Microbial Communities Residing in Arid and Ephemerally Wet Antarctic Dry Valley Soils
Source: Front Microbiol. 2015 Dec 9;6:1347. doi: 10.3389/fmicb.2015.01347 (PMC4673872; doi:10.3389/fmicb.2015.01347)
Supplement: Supplementary file 4 [file Data_Sheet_2.DOCX]

**Figure 2.** Rarefaction at 97% and 95% similarity of 16S rRNA genes from wet (ML1-2) and arid (ML1-4) soil sites.
