# Supplementary material for: Carbon-Fixation Rates and Associated Microbial Communities Residing in Arid and Ephemerally Wet Antarctic Dry Valley Soils
Source: Front Microbiol. 2015 Dec 9;6:1347. doi: 10.3389/fmicb.2015.01347 (PMC4673872; doi:10.3389/fmicb.2015.01347)

**Figure 3a.** Rarefaction analyses of 18S rRNA genes at 90 and 95% similarity from wet (ML1-2) soil.


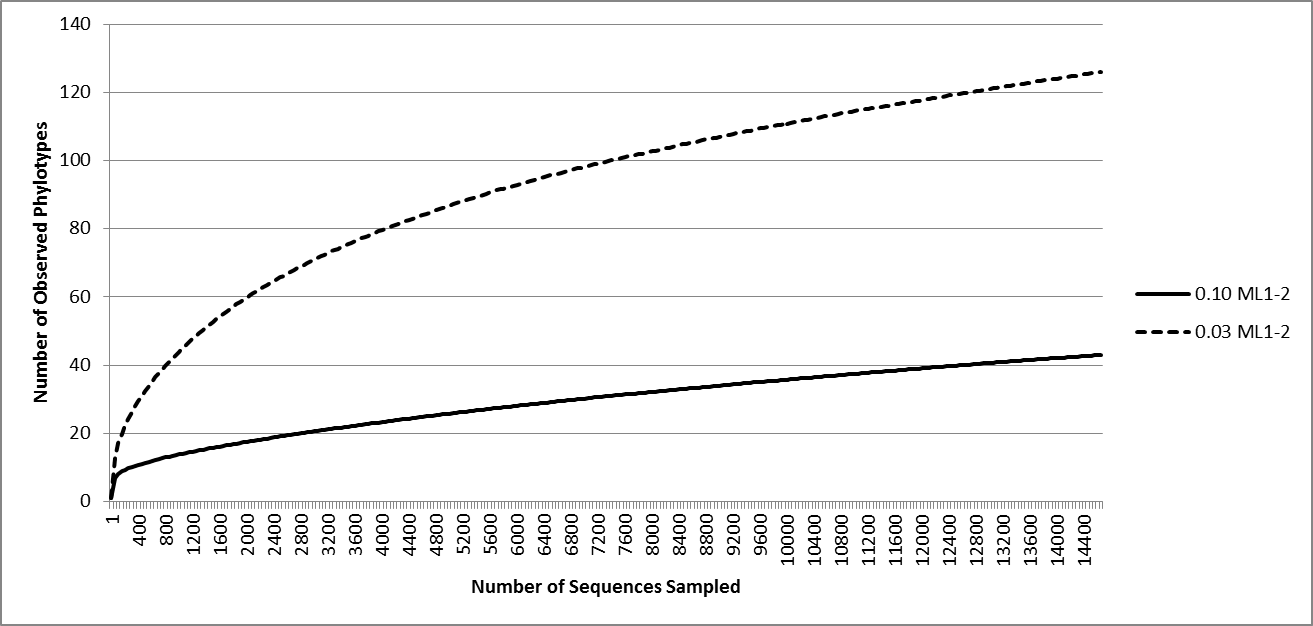


**Figure 3b.** Rarefaction analyses of 18S rRNA genes at 90 and 95% similarity from arid (ML1-4) soil.


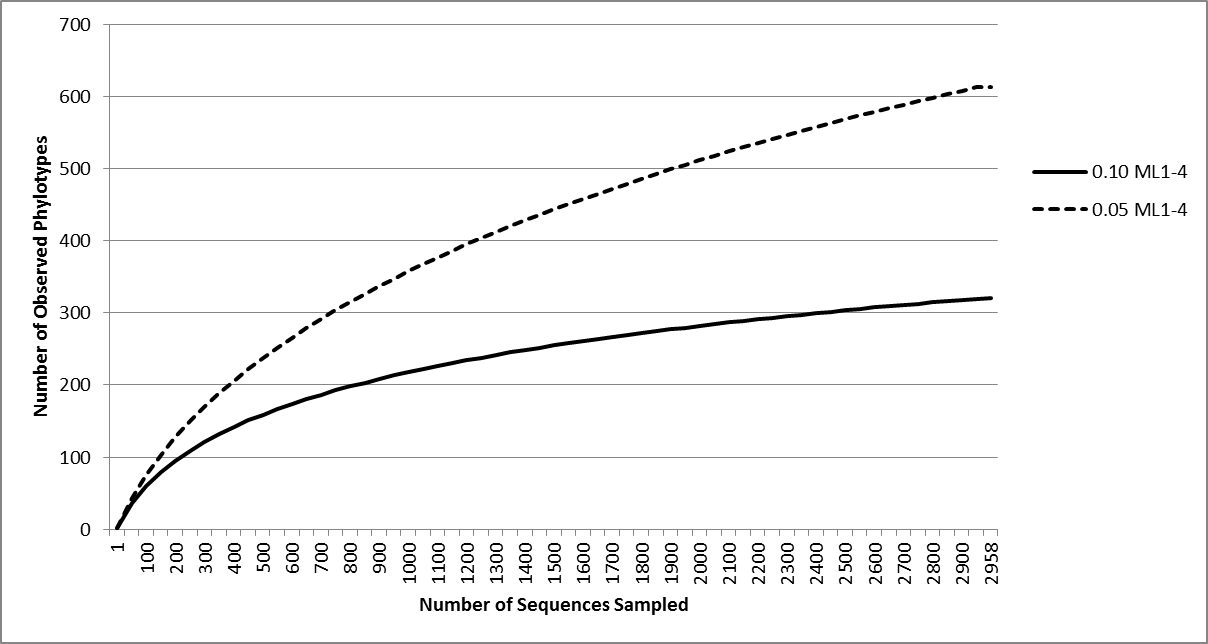

Supplement: Supplementary file 5 [file Data_Sheet_3.DOCX]
